# Food Polyphenols as Preventive Medicine

**DOI:** 10.3390/antiox12122103

**Published:** 2023-12-12

**Authors:** Joseph Kanner

**Affiliations:** 1 Department of Food Science, ARO, Volcani Center, Bet-Dagan 7505101, Israel; joseph.kanner@mail.huji.ac.il or jokanner@gmail.com; 2 Institute of Biochemistry, Food Science and Nutrtion, Faculty of Agriculture Food and Environment, The Hebrew University of Jerusalem, Rehovot 9190501, Israel

**Keywords:** diet, stomach, lipid peroxidation, reactive carbonyls, timing, antioxidants, polyphenols, signaling factors, NF-κB, Nrf2, eustress

## Abstract

Reactive oxygen species (ROS) are the initiators in foods and in the stomach of oxidized dietary lipids, proteins, and lipid-oxidation end-products (ALEs), inducing in humans the development of several chronic diseases and cancer. Epidemiological, human clinical and animal studies supported the role of dietary polyphenols and derivatives in prevention of development of such chronic diseases. There is much evidence that polyphenols/derivatives at the right timing and concentration, which is critical, acts mostly in the aerobic stomach and generally in the gastrointestinal tract as reducing agents, scavengers of free radicals, trappers of reactive carbonyls, modulators of enzyme activity, generators of beneficial gut microbiota and effectors of cellular signaling. In the blood system, at low concentration, they act as generators of electrophiles and low concentration of H_2_O_2_, acting mostly as cellular signaling, activating the PI3K/Akt-mediated Nrf2/eNOS pathways and inhibiting the inflammatory transcription factor NF-κB, inducing the cells, organs and organism for eustress, adaptation and surviving.

## 1. Introduction

Epidemiological, clinical and animal studies supported a role of polyphenols in the prevention of chronic diseases such gastrointestinal diseases (GITD), cardiovascular disease (CVD), diabetes, neurodegenerative diseases and cancer. The health benefit of the Mediterranean diet is attributed to the high consumption of foods containing polyphenols. Paradoxically, polyphenols are barely absorbed in the gastrointestinal tract (GIT) and they undergo extensive metabolism in the GIT lumen, enterocyte and liver. The low absorption and concentration of these compounds and metabolites in blood plasma and peripheral tissues does not support their activity in blood as competitive reducing antioxidants and their mechanism of activity in human is not fully understood. In order to understand the reactivity of polyphenols as preventive medicine compounds we need to understand the chemistry of these important constituents present in our diet.

Polyphenols are plant secondary metabolites exhibiting central functions in plant protection against various biotic and abiotic stresses by their potential to activate signaling factors and act as antioxidants and cytotoxic agents. In plants they have antimicrobial, antiviral, antifungal, anti-insect, anti-herbivore, wound-healing, drought and UV protection properties [1]. Polyphenols are naturally occurring compounds present in fruits, vegetables, spices, and beverages, and are classified into several classes that include phenylpropanoides and they number more than 10,000 in plants. Phenolic compounds are those that have at least one benzoic ring with one or more hydroxyl groups. The amino acid phenylalanine is the precursor to all polyphenols which are biosynthesized by specific enzymes to non-flavonoids such as phenolic acids (gallic acid), and flavonoids such as flavonols (quercetin), flavonals (catechin) and anthocyanins (cyanidin). A part of the flavonoids is condensed to tannins such as procyanidins and proanthocyanidins. Hydrolyzable-tannins (gallotannins or ellagitannins) are polymers readily hydrolyzed into their components, glucose, flavonoid and carboxylic acid [2].

Many reviewers describe the activity of specific polyphenols such as, quercetin, catechin, caffeic acid, cyanidin, etc., on human health without offering a general mechanism which may explain polyphenol’s synergistic activity in an organism. Different food polyphenols in the gut and blood system act in unison and almost in the same way. In the stomach and different parts of the gut at high-concentration, all of them act as antioxidants, but in the cardiovascular system (CVS) at low concentration, polyphenols/derivatives act mostly as pro-oxidants. All polyphenols interact with proteins/enzymes, because the benzoic group hydroxyls form with amino acids hydrogen bonds, affecting digestion, microbiome growth and activity. Most polyphenols/derivatives activate at the level of endothelial cells signaling factors, by low concentration of H_2_O_2_, generated at the surface of the cells and diffuse in cells through aquaporins [2].

The aim of this review was to collect data on the activity of various polyphenols in vitro and in vivo to affect human health and to propose a general mode of action for polyphenols in organs such as, the stomach, gastrointestinal tract, colon, cardiovascular system, affecting the liver, pancreas, kidney, and brain, causing the organism to eustress and thus, prevent disease.

## 2. Polyphenols as Reducing Agents and Antioxidants

Compounds which reduce the rate generation of reactive oxygen species (ROS), lipid free radicals and hydroperoxides act as antioxidants [3]. Several mechanisms behind such effects have been found and these allow to classify antioxidants in four major classes: (a) reducers of radical initiators and peroxides; examples for the first class are enzymes which destroy superoxide (superoxide dismutases), hydrogen peroxide (peroxiredoxins, thioredoxins, peroxidases, and catalase), hydroperoxidases (peroxidases or hemeproteins coupled with glutathione, thiols, polyphenols, or ascorbic acid); (b) chain breaking reducers (tocopherols, tocotrienols, polyphenols); (c) metal catalyzer’s chelators (citric acid, polyphenols); and (d) singlet oxygen quenchers (carotenoids). Polyphenols and tocopherols belong to all classes of antioxidants; (1) as reducers of radical initiators, polyphenols act as “coenzymes”, with peroxidases and hemeproteins recycling the oxidized form of the enzyme/hemeprotein to the reduced form. By such activities, polyphenols help to break-down hydrogen peroxide and hydroperoxides catalytically to non-radical forms, preventing the autocatalytic destruction of the enzyme/hemeprotein to non-active forms. (2) Polyphenols act mainly as chain-breaking free-radicals. The antioxidant activity of phenolic compounds is best evaluated by determination of the rate constants for H-atom abstraction or electron donation from the phenolic groups by a radical such as peroxyl radical. (3) Polyphenols are metal chelators, by this activity they can act as antioxidants or pro-oxidants. (4) Polyphenols are hydrogen-bonders, this reactivity increases their interaction with many molecules and especially with proteins such as enzymes, substrates, receptors and many other active molecules changing their activity. (5) Polyphenols are good quenchers of singlet oxygen.

Active polyphenols owe their activity to a combination of electronic and steric effects which lower the bond dissociation enthalpy of the O-H bond which increases its reaction with phenoxyl, peroxyl, alkoxyl radicals. The intra-molecular or inter-molecular H-bonding to the O-H groups around the polyphenols increases also the tautomeric effects generating resonance to four forms of the phenol molecule [4,5]. One, the regular structure and other three hydroquinone cation radicals (HQ+) containing an un-paired electron on ortho, meta or para positions of the benzene ring [2], (see Reactions (1)–(7))

Ph-OH + electrophiles/M^(+n)^/R^•^→ Ph-O^•^ + reduced compounds (1)

Ph-O^•^ + O_2_ → Ph=O + O_2_^•−^ + H^+^ (2)

Ph-OH + O_2_^•−^ + H^+^ → Ph-O^•^ + H_2_O_2_ (3)

Ph-O^•^ + O_2_^•−^ + 2H^+^ → Ph=O + H_2_O_2_ (4)

Ph-OH + Ph=O → 2 Ph-O^•^ (5)

Ph-O^•^ + ArOH → Ph-OH + Ar-O^•^ (6)

HQ^+•^ + O_2_ → Ph=O + H_2_O_2_ (7)

where: Ph-OH = reduced polyphenols; Ph-O^•^ = semiquinone-phenoxyl radical; Ar-O^•^ = Aroxyl radical: O_2_^•−^ = superoxide radical; Ph=O = quinone; M^(+n)^ = oxidized metal ion; R^•^ = free radical, HQ^+•^ = hydroquinone cation radicals.

The hydroquinone cation radical structure acts to lower the bond dissociation enthalpy of the O-H bond increasing the reducing capability of the molecule. There is considerable evidence that intramolecular H bonding, such as in the catechol or better in the galloyl form of the ring system, has a pronounced effect on the reducing activity. The optimal reducing activity of polyphenols (including flavonoids) is governed mostly by three criteria originally proposed by Bors and associates [6]: (1) The presence of the ortho-diphenol/galloyl structures at the B-ring: (2) The presence of the 2, 3 double bond in conjugation with a 4-carbonyl group at heterocyclic ring C: (3) The presence of hydroxyls at position 3 and 5 of rings C and A, respectively. These criteria were found to be correct by determining the reducing activities of polyphenols at pH 3 in a simulated stomach medium [7]. The hydroxyl groups act also as a weak acid. The pH strongly affects the activation energy needed for the displacement of the electron from the phenol group. This activation energy decreases by increasing the pH, thus forming a phenolate which better donates the electrons. The scavenging of a free radical by the polyphenol gave rise to a phenoxyl radical. The resulting phenoxyl radical must be sufficiently stable or in a redox potential which does not initiate a new chain reaction. One of the features that stabilized the phenoxyl radical was the aromatic structure of the benzene ring that allowed the formation of aroxyl radicals by resonance. The reaction between a phenoxyl with a peroxyl radical is less rapid than between a phenoxyl radical with an aroxyl, thus generating a phenoxyl and aroxyl radical [8]. This reaction is easily adjusted when polyphenols are in a very broad mixture such as in natural plant material generating a synergistic antioxidant effect by the Reaction (6).

The electronic configuration of the hydroxyls around the benzene ring affects not only the redox activity but also the metal chelation reactivity. The non-bonded electrons around the hydroxyls could form coordinative bonds with many transition metals such as iron, copper and others which act as catalyzers of lipid oxidation. This sequestration of metal ions decreases lipid oxidation but paradoxically, could also enhance it. If the polyphenol is of small molecular weight and at relative low concentration, it mostly enhances lipid oxidation by an “iron redox cycle” pathway. However, if they are of great molecular weight with many hydroxyls and hydrophobic groups, at the same concentration, it inhibits lipid peroxidation. This effect arose due to the high molecular weight of polyphenol containing more hydroxyls, its redox is higher, and thus acts better to displace the metal ion from the fatty acids. The chelated and reduced metal ion will breakdown hydroperoxides to alkoxyl radical which will interact with the phenoxyl radical to a non-radical adduct preventing generation of free lipid radicals. At a critical high-concentration and in greater excess to metal ions or iron hemeproteins, polyphenols could act together with the metals via a peroxidase-like activity to catalyze the breakdown of H_2_O_2_ and ROOH to water or hydroxyl fatty-acids, inducing stabilization of the system toward lipid peroxidation, without forming cytotoxic reactive aldehydes or others toxins [7]. Kinetic aspects should be considered for the reducing effects of polyphenols because oxidation and anti-oxidation in biological systems are very much affected by metal or enzyme catalysis, membrane structure, molecule solubility and polarity, pH, water-activity, bioavailability, and metabolism. For these reasons determination of an antioxidant activity in a biological, food or nutrient system should be not only a test but a thorough study [2].

## 3. Polyphenols as Pro-Oxidants

The electronic configuration of the hydroquinone cation radical containing an unpaired electron around the benzene ring opens the possibility of direct interaction with ^3^O_2_ oxygen (Reaction (7)). The tautomeric effect generating the hydroquinone cation radicals allow them for direct interaction with ^3^O_2_ forming super-oxide anion radical (O_2_^•−^) perhydroxyl radicals (HO_2_), hydrogen peroxide (H_2_O_2_), semi-quinones) Ph-O^•^) and quinones(Ph=O) [2].

The generation of quinones in the presence of reduced polyphenols and ^3^O_2_ initiates the pathway for auto-oxidation of polyphenols. One possible route of these electrophiles in the organism at very low concentration is the activation of transcription factors [2]. At a relatively high concentration of polyphenols the pro-oxidative route is cytotoxic capable of modifying proteins and DNA [9,10,11,12,13]. The geometric isomers of hydroquinones for activation to electrophiles are important, because only the ortho and para hydroquinones and not the meta-forms are active to generate electrophiles [14]. The capability of polyphenols to act as chelating agents allow them to affect the allocation of transition metals such as iron, copper, zinc and others in the cells and out of the cell and act together as a couple which enhance oxygen activation to free radicals. Chelating agents such as diethyl-dithiocarbamate was found by allocation of copper ions from liver mice cells to enhance the polyphenol copper redox-reaction and the cytotoxic effects of high concentration of polyphenols in organs such as mouse liver [12]. These interactions between polyphenols and derivatives allowed them to act at the same time as reducing agents-antioxidants and generators of active oxygen species.

## 4. Polyphenols and the GI Tract

The non-bonded electrons around the polyphenol hydroxyls could not only form coordinative bonds with transition metals but also form hydrogen bonding with molecules such as proteins (enzymes, receptors, signaling factors) and by these affecting gut enzyme activity, endogenous cell transcription, biosynthesis, and microbiome activity [2,15]. Polyphenols in GIT act at the same time as reducing agents/antioxidants and as generators of H_2_O_2_ and other reactive species. H_2_O_2_ generated on the cells membranous area (because of the high affinity of polyphenols to membranes) after penetration into cells using aquaporin, at nM concentration, affects cell signaling and transcriptional generation of protective and adaptive proteins to oxidized stress [2]. Recent research suggests that these polyphenols in human diet are no longer mere radical scavengers but rather modulators of gut microbiota composition and cell signaling [16]. However, this review will present much evidence that these compounds act in the stomach and generally in GIT as very important reducing agents, free radical scavengers, modulators of enzyme activity, gut microbiota composition and cell signaling.

### 4.1. Mouth 

Saliva has multiple roles in relation to the GI, such as bolus formation, enzymatic digestion, decreasing astringency and buffering. It is also involved in the immunological defense system, which is based on protein enzymatic defense process and the secreted immunoglobulins. Another significant function of saliva has been discovered to act as an antioxidant system. Saliva mainly contains low-molecular-weight antioxidants such as uric acid, ascorbic acid, glutathione, thiocyanate and antioxidants from the nutrients and especially polyphenols [17]. The maintenance of an appropriate redox status in the oral cavity is crucial to prevent adverse effects from high-concentrations of reactive oxygen species such as H_2_O_2_, O_2_^•−^, and peroxynitrite.

All types of oxidants generated in the oral cavity might be involved in cell and tissue injury. Ginsburg et al. [18] found that saliva has an important role of increasing polyphenol antioxidant activity in the mouth. The persistence of the polyphenols in the saliva could favor their antimicrobial activity against some oral bacteria, preventing the formation of pathogen biofilm in the tooth surface [19]. Dietary nitrate is concentrated in the saliva and reduced to nitrite by oral flora. The salivary nitrite concentration ranges from 100 to 150 µM (6–9 mg/L) but it could increase to 1400 µM (~100 mg/L) following a high-nitrate meal [20]. In the stomach, nitrite saliva mixes with food, drink and gastric fluid is diluted by a factor of 10–20 to ~15 µM. Nitrite at low pH is decomposed to nitrous acid, N_2_O_3_, a nitrosating agent forming with secondary amines nitrosamines which are carcinogenic compounds; however, in the presence of polyphenols or other strong reducing compounds, nitrite mostly decomposes to NO [17,21].

### 4.2. Stomach

The stomach, which acts as a bioreactor, is an important intersection in human for chemical and biochemical reaction and an excellent medium for further food/meat lipid peroxidation [22,23], generation of more hydroperoxides, co-oxidation of dietary constituents such as cholesterol to cholesterol oxidized products (COP) [24], co-oxidation of vitamins [25], generation of cytotoxic carbonyls and advanced lipid oxidation end-products (ALEs) [26,27,28]. Lipid peroxidation of red meat in stomach medium was increased tremendously by fish oils and reduced in the presence of olive oil [29]. Many of the products generated from lipid hydroperoxides breakdown, such as reactive aldehydes, ketones, and epoxides are cytotoxic [27,30,31,32]. Once formed in the stomach they interact with dietary proteins to ALEs, which breakdown in the intestine by proteases to amino-acid-carbonyls which are readily absorbed into the blood system, and subsequently interact with functional proteins and lipids to form secondary advanced lipid oxidation end-products (ALEsII) [33,34]. ALEsII and AGEsII in the blood system interact with RAGE (receptor of advanced glucose end products) to activate a postprandial oxidative stress [35,36,37,38,39]. Animals and humans, after ingestion of peroxidized foods, have been shown to absorb and excrete an increased amount of malondialdehyde (MDA) and other carbonyls [40,41,42]. Polyphenols and foods rich in polyphenols were found to inhibit food lipid peroxidation in stomach medium co-oxidation of vitamins and cholesterol and production of primary and secondary ALEs [26]. These results were supported by several clinical studies found that polyphenols and foods rich in polyphenols could prevent food lipid peroxidation in the stomach absorption and accumulation of reactive carbonyls in pig and human blood systems or the modification of LDL [42,43,44,45,46,47]. We suggest that the main benefit of consuming plant polyphenols and other redox compounds in the human diet, as an integral part of the meal, arises mainly from the ability to prevent lipid peroxidation, co-oxidation of vitamins, generation of reactive aldehydes and other cytotoxic ALEs in the stomach. Due to this action, polyphenols decrease the absorption of reactive aldehydes and other cytotoxic compounds into the blood system, and increase the bioavailability of vitamins. Both factors act synergistically for improving human health. We presented a study which used simulated gastric fluids (SGF) to assess the capacity of more than 50 food products of plant origin to suppress red meat peroxidation and the formation of carbonyls in stomach medium. The results were calculated into a Postprandial Oxidative Stress Index (POSI). The reduction POS index (rPOSI) represents the capacity of the food used to inhibit lipid peroxidation of meats by POS enhancers (ePOSI) [48] (see Table 1). The index permitted to extrapolate the need of rPOSI from a single food alone or in a combination such as a salad, to neutralize an ePOSI in stomach medium, (ePOSI − rPOSI) = 0(POSI). The correlation between the rPOSI and fruits polyphenols in the tested foods was R^2^ = 0.87. The Index was validated by testing the predicted rPOSI for a food (vegetable salad or red-wine) portions to its real inhibition of red-meat lipid peroxidation and was found to be highly predicable (see Table 2). It was found that a person should ingest ~150–200 mg of polyphenols (from fruits, vegetables, or beverages) during a meal to inhibit lipid peroxidation in stomach by 200 g of red meat. Keeping the redox homeostasis in stomach medium, by foods and beverages during the meal, seems to be an important nutrition factor for healthy living. On an equal caloric basis and a right timing and dosage consumption the POS Index should help to turn the Western diet pattern to a Mediterranean one for better balancing nutrition and human health [48].

Cholesterol oxidation products (COP) are generated in foods during co-oxidation with free radicals’ fatty-acids. Cholesterol oxidation via lipid free radicals results in the formation of many oxidation by-products such as 7α and 7β hydroxyl-cholesterol, 7-keto-cholesterol, and 5α and 6α epoxy-cholesterol, all of them pro-inflammatory and pro-apoptotic agents in human [15,27,49,50]. COP has been found and quantitated in cholesterol rich processed foods. They are particularly prevalent in food products such as dried egg, milk powders, heated butter-ghee, but also in precooked meat and poultry products. The amount of COP in these products could reach 10 to 100 µM; in ghee the amount of COP exceeded 12% of the cholesterol found in butter [27]. The potential role of COP in human pathogenesis increases from the recent hypotheses on the developing of chronic diseases that are affected by oxidative distress and inflammation, such as atherosclerosis, Alzheimer’s disease and inflammatory bowel disease [49,51]. Red wine and tea polyphenols were found to inhibit COP generation in stomach medium or intestine, and their increase in human plasma after a meal containing a cheeseburger [52].

Nitrite produced in the mouth saliva from dietary nitrate reaches the stomach during swallowing. In stomach nitrite can react with secondary amines at the low pH of gastric fluid to form nitrosamines [53]. The acidification of nitrite in the stomach results in the formation of nitrous acid and nitrogen oxides by the following reactions:

NO_2_^−^ + H^+^ → HNO_2_ (8)

2HNO_2_ → ^•^NO + ^•^NO_2_ + H_2_O (9)

^•^NO + ^•^NO_2_ ↔ N_2_O_3_ (10)

The interaction between nitrous acid and nitrogen oxides with secondary amines (R_2_NH) generates nitrosamines via the following reaction.

HNO_2_ + R_2_NH → R_2_N-NO + H_2_O (11)

N_2_O_3_ → ^•^NO + ^•^NO_2_ + R_2_NH → R_2_N-NO + HNO_2_ (12)

Nitrite has been traditionally used by the food industry as an additive to serve a multifold purpose; (1) preservation of meat, owing to the unique ability of nitrite to prevent the growth of *Clostridium botulinum* [54]; (2) development of the characteristic pink-red color of the meat [55]; and (3) contribution to lipid and flavor stability [56]. The mechanism of lipid and flavor stability was found to be affected due generation of antioxidants such as myoglobin-NO, cysteine-NO and NO radicals, which prevent lipid peroxidation [57,58,59]. The World Health Organization has issued a warning regarding the consumption of nitrite processed meat, indicating its definite carcinogenic effect in humans. Additionally, a warning was also issued regarding consumption of high amounts of red meat, in which its over consumption also provides a probable carcinogen [60]. Details on this decision were published in the Lancet Oncology [61]. In 1972 Mirvish et al. reported that sodium ascorbate as a reducing agent inhibited the formation of nitrosamines in vitro because at low pH it reduced nitrite to NO but also because ascorbate could scavenge N_2_O_3_ (or ^•^NO + ^•^NO_2_ radicals) preventing nitrosation of amines [62]. Meanwhile, ascorbic acid, which is actively secreted in the stomach, is a major inhibitor of acid catalyzed nitrosation in the stomach lumen [63,64,65]. The potential chemoprotective effect of phenolic compounds against nitrosative stress in stomach has been highlighted in the past [66,67,68]. Gallic, caffeic and ferulic acids were effective of preventing nitrosation of R_2_NH but less than ascorbic acid [69]. Both ascorbate and gallic acid were found to decrease significantly mouse lung adenoma induced by a diet of amines and nitrite [70]. Fruits and vegetable juices were found to decrease endogenous formation of N-nitrosoproline in human on controlled diets [71]. As NO generated from nitrite in the presence of oxygen could form new oxidizing nitrogen oxides enhancing nitrosation, Combet et al. [72] identified reducing agents which inhibit nitrosation from nitrite in stomach conditions without generating high concentration of NO. We found that nitrite in stomach under aerobic conditions oxidizes beta-carotene ~10-fold more rapid than NO, (most probably by NO derivatives). The addition of reducing agents such as ascorbic acid, catechin or red wine polyphenols inhibited nitrite oxidation of beta carotene in simulated stomach fluids. NO is a well-known promotor of vasodilation and has an essential role in cardiovascular health, protecting against ischemia-reperfusion injury and inhibiting platelet aggregation [73]. In stomach conditions polyphenols reduced nitrite to NO [74]. Patients with metabolic syndrome showed sustained improvements in vascular function, lipid status and underlying NO bioactivity following an intake of one cup of blueberries (containing a high concentration of polyphenols) per day for 6 months [75].

### 4.3. Intestine

Polyphenols in the intestine could affect other biochemical important reactions such those which are related to the digestion of proteins, carbohydrates, and lipids. Polyphenols act as hydrogen bonders and hydrophobic association effectors could affect protein digestibility in three different ways. (a) Interaction with dietary proteins. (b) Interaction with the active site of gut-enzymes. (c) Interaction with gastric and intestinal mucus cells. A large amount of papers were published on these areas [76,77,78,79]; most of the authors found that polyphenols decrease nutrients bioavailability. In developing countries, the diet is predominantly based on less refined cereals and legumes. These products contain less digestible protein due to the presence of high amounts of anti-nutritional factors, including polyphenol compounds. Food products such as sorghum, millet, and beans contain considerable amounts of tannins, ~700 mg/100 g, which very much affect the digestibility of proteins and reduce the quality of these foods [80]. Several well-known technological treatments have been developed to reduce the number of polyphenols in these foods, such as soaking in water, soaking in water and then in hot water, soaking in alkaline solutions, heat treatment with or without high pressure, roasting, de-hulling, fermentation, germination, and bonding to chemicals with a high affinity for polyphenols and polyphenols-proteins aggregates. There are several plant materials containing a huge number of polyphenols, such as green olives, coffee, cocoa beans, and tea leaves, which before production as foods (with specific color, aroma, texture, or nutritional value) the industry developed special processing methods to decrease or change their molecular composition. Green olives contain such high amounts of polyphenols that they prevent olive fermentation by lactic bacteria [81]. It is a “Spanish method” to ferment the olives only after a treatment of fruit peel with NaOH, which breakdown the ester-bonds in peel-waxes, affecting peel wholeness and permitting the polyphenols washing out.

Polyphenolic extracts from plant origin were reported to act as effective inhibitor of saliva α-amylase and intestinal α-glucosidase, sucrose iso-maltase, maltase, in vitro [82] and in vivo [83]. Carbohydrate digestion can be attenuated by inhibition of enzymes which decompose saccharides to mono-saccharides and prevent them transport across the enterocytes [84]. Polyphenols also affect the active sodium-glucose co-transporters1 (SGLT1) and glucose transporter (GLUT5 and GLUT2). However, one should take in consideration, that in meals containing large concentration of proteins, the inhibition of enzymes and transporters by polyphenols could decrease considerably due the hydrophobic/hydrophilic competition by dietary proteins [77,84].

Like other enzymes and transporters, polyphenols affect also enzymes which are involved in lipid digestion and absorption. Most of the studies have been focused on the inhibition of pancreatic lipase (PL), phospholipase A2 (PLA2) and cholesterol ester hydrolase (CSH) [76,85,86]. The amount of the hydroxyl groups in polyphenols was found to positively correlate with PL inhibition [87]. The presence and location of the galloyl groups were essential for PL inhibition by tea theaflavins [88]. From all the articles published on the mechanism of the enzyme inhibition by polyphenols the importance of hydroxyls, hydrogen bonds and hydrophobic competition is evidential. Due to this molecular structure, polyphenols know to interact in intestine with electrophilic reactive carbonyls, preventing generation of ALEsII/AGEsII, and reducing these compounds absorption from the GIT lumen to the blood system [89,90].

Increasing evidence links intestinal permeability (IP), a feature of the intestinal barrier, to several pathological or dysfunctional conditions. Several host and environmental factors, including dietary factors, can affect the maintenance of normal IP. In this regard polyphenols have been proposed to serve as potential IP modulators, even if the mechanisms involved are not yet fully elucidated [91]. IP evaluation can be used to address a normal/stable or disturbed/compromised permeability related with intestinal barrier function. Intestinal barrier integrity and functionality can be affected also by the characteristics of the intestinal microbial ecosystem and mucosal immune system. It has been demonstrated that the Western diet, characterized by high-energy and high-fat intake or high-fructose consumption, can alter IP by affecting the gut microbiota composition [92]. The Mediterranean diet, rich in fruits, vegetables, legumes, and unrefined cereals rich in polyphenols has been suggested to positively affect IP [93].

Polyphenols may exert their effects by down-regulating inflammatory signaling factors such as nuclear factor kappa B) NF-κB) and upregulating cyto-protective signaling of nuclear factor 2 (Nrf2) [2,94]. This modulation may bring a reduction of cytokine production [e.g., interleukin (IL)-8, IL-1β, and tumor necrosing factor alpha (TNF-α)] and boost the cells own antioxidant status of heme oxygenase 1 (HO-1), superoxide dismutases (SODs), and glutathione peroxidase (GPx)] [95]. Furthermore, reviews, ref. [96,97] have been shown that polyphenols may affect, in either a positive or negative way, pattern recognition receptors, such as toll-like receptors (TLR) and nucleotide-binding oligomerization domain proteins, whose activation in epithelial cells may affect IP.

Some studies hypothesized a direct/indirect involvement of (NF-κB) signaling in the onset of IP. The putative effects of polyphenols on IP at different physiological levels are affected by: (1) intraluminal level: modulation of microbiota composition, endotoxin and/or short chain fatty acids (SCFA) production, redox status, and dietary component absorption and/or activity; (2) intracellular level: regulation of expression of tight junctions (TJ), adherence junction (AJ), gap junction (GJ), and desmosome proteins, upregulation of kinases and Nrf-2, and down-regulation of NF-κB and TLR4; and (3) systemic level: maintenance of the functional immune system and regulation of inflammatory processes (toward a reduced pro-inflammatory status) [91]. Other important factors potentially involved in increasing IP are the multiple protein kinases, such as MAPK, phosphoinositide-3-kinase/protein kinaseB (PI3K/Akt), protein kinase C (PKC), tyrosine kinase, myosine light chain kinase (MLCK), and adenosine monophosphate (AMP)-activated protein kinase (AMPK). Most of them are regulators of fundamental biological processes in epithelial cells, including barrier function, primarily through regulating TJ expression. Some polyphenols (e.g., quercetin, curcumin, epigallocathechin 3-gallate, and myricetin) have been shown to improve the epithelial barrier function through the inhibition of different kinases (PKC and MLCK) involved in phosphorylation of target proteins controlling IP [98,99,100]. Some other polyphenols such as resveratrol and ellagic acid were also found to affect IP against oxidative stress affected by H_2_O_2_ or TNFα in IPEC-12 and Caco-2 cells, respectively [101,102]. Resveratrol attenuates oxidative stress induced intestinal barrier injury and IP through a PI3K/Akt mediated Nrf2 signaling pathway [103]. Ellagic acid and cyanidin protect intestinal cells against inflammation induced IP due inhibition of NF-κB and 1/2 (ERK1/2), most probably by activation of Nrf2 [104]. Several anthocyanins were determined for the inhibition of TNFα-induced loss of intestinal cell barrier integrity. The results show that the most active compounds were those with two or more hydroxyls on anthocyanin-B ring which are known to better auto-oxidize and produce more H_2_O_2_ [2,105]. The results support the capacity of cyanidins and delphinidins in the protection of the intestinal barrier against inflammation induced IP due to inhibition of the NF-κB pathway and activation of the Nrf2 axis [106]. It could be hypothesized that most those effects on IP derived indirectly from polyphenols by generation a low H_2_O_2_ concentration on epithelial cell surface [2].

Most of the studies in animals on IP were induced by stimuli, such as high-fat diets, mannitol, inflammatory cytokines, or chemicals [91,107,108,109]. Inflammatory cytokines increase the production of RAGEs in intestine and colon cells making the cells more vulnerable to external dietary ALEs/AGEs and in general to oxidative stress and dysbiosis [110]. The main polyphenols used to protect IP were obtained from grape seed extracts and grape seed proanthocyanidin extracts, berberine, epicatechin and epigallocatechin-3-gallate [91]. The studies showed the capacity of polyphenols to upregulate some important genes generating kinases such as AMPK and ERK, and downregulate NF-κB, pathways involved in the inflammation process. In line with the observations reported in the in vitro cell studies, the compounds tested have shown to increase the expression of Zonula occludins-1 antibody (ZO-1), and several claudins involved in the functioning of TJ. The results from in vitro studies have shown the capacity of polyphenols to increase the expression and/or production of numerous TJ proteins and to reduce the release of several cytokines.

### 4.4. Colon

Animal and human studies show that polyphenols have dramatic effects on human gut microbiome. These compounds appear to modulate both species composition within the gut microbiome and the profile of metabolites generated from polyphenols by the microbiome and absorbed from the colon into the blood system. Dietary fibers, which also greatly affect gut microbiome, were found to contain ~20% and more of non-extractable polyphenols [111], arrive at the colon and affect the microbiome. Flavanols induced changes to human microbiome, changes which were obtained in a human study using cocoa-derived flavanols [112], and red and white grape polyphenols [113]. Most of the studies resulted in significant increases in fecal bifidobacteria and lactobacilli and a reduction in non-beneficial clostridium count. Similar results were obtained by using tea polyphenols [114], or breakfast cereal polyphenols [115,116]. It was found that in healthy adults who consumed only vegetable/fruit juices, the proportion of the phylum Firmicutes and Proteobacteria in stool was significantly decreased and bacteroidetes and cyanobacteria increased by day 4 [113]. Flavonoids are commonly ingested diet-derived compounds that are metabolized by intestinal microbiota. Such a combination of dietary flavonoid available and microbiome-mediated flavonoid degrading capacity may contribute to the total intestinal flavonoid pool. Gut microbiome catabolism of polyphenols include, (a) hydrolysis of ß-glucosides to form phenolic-aglycones, containing benzoic ring-A; (b) ring-cleavage and methylation products; (c) reduction reactions which de-hydroxylate and de-hydrogenate, producing most of the compounds which are in part absorbed into the blood system, such as 2–3 di-hydroxy-phenyl propionic acid, hydroxyl-phenyl propionic acid, equol, urolithin A and B, and many other phenolic acids with phenol or catechol hydroxyls [117,118]. Urolithin A, a major microbial metabolite derived from polyphenols such as proanthocyanidins or ellagitannins, displays anti-inflammatory functions, a significantly enhanced gut barrier function in vitro and in mice. It was demonstrated that Urolithin A exerts their barrier functions through activation of aryl-hydrocarbon-receptor and generation of Cytochrome 1A1 (Cyp1A1). Cyp1A1 once more activates Urolithin A, most probably by hydroxylation to an active form of polyphenol which activates Nrf2, and upregulates epithelial TJ proteins and cell adaptation [94]. These results are partially in line with the findings obtained in the animals and human models, showing the capacity of polyphenols to up-/downregulate some important genes involved in the inflammatory process and generation of proteins acting for cell and organ oxidative eustress adaptation and surviving [2,119,120].

A high fat diet including unsaturated fatty acids and trans-fatty acids can increase the IP and contribute to an increase in intestinal permeability and increase in gut-bacteria-derived lipopolysaccharide (LPS). LPS binds to TLR4 and initiates a cell inflammatory response. Foods rich in polyphenols and prebiotic fibers (mostly containing non-soluble polyphenols) can favor short-chain fatty-acids (SCFA) production bacteria and beneficially modulate gut microbial composition and function. SCFAs attenuate inflammation in a free fatty-acid receptor-dependent manner, whereas phenolic metabolite containing hydroxyls and catechol-benzene compounds, by generation of H_2_O_2_, upregulate the Nrf2 axis and attenuate cells oxidative distress [2]. HFD was found to promote the growth of flavonoid-metabolizing bacteria, which in turn decreases the number of bioavailable flavonoids which are important to ameliorate post-dieting obesity. Interestingly, weight-adjusted energy expenditure was markedly reduced in weight-cycling mice, but was normalized upon flavonoid administration [121,122,123]. The research shown that apigenin and naringenin (and not the catabolized flavonoid compounds) after two weeks of administration elevated significantly the factor uncoupling protein-1 (UCP1) transcript levels in the brown adipose tissue (BAT) of mice fed the HFD [121,122,123]. BAT is a major regulator of thermogenesis in mammals. Since other flavonoids (quercetin, hesperetin, epicatechin, apigenin, blackcurrant anthocyanins, theaflavins, chrysin) have previously been associated with the induction of the major thermogenic UCP1 in BAT [124,125,126], it seems that this is an important pathway by which flavonoids may affect overweight individuals. The elevation of UCP1 is also induced by H_2_O_2_, most possibly generated by auto-oxidation of polyphenols [2,127].

**Figure 1:**
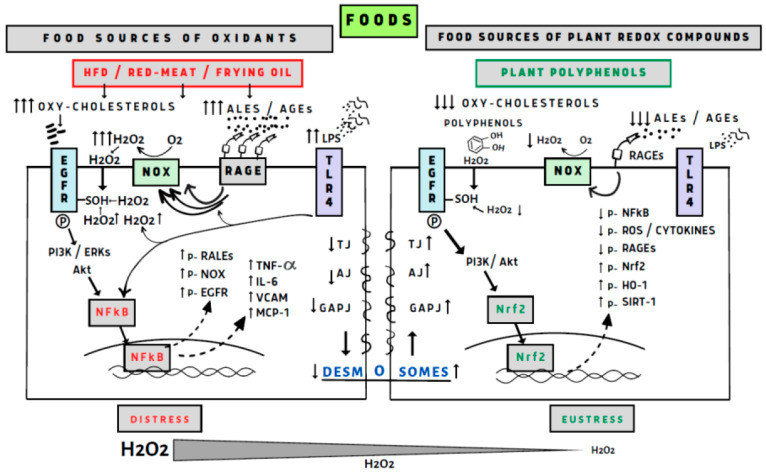
The effect of dietary oxidants and polyphenol antioxidants on cellular gastrointestinal (GI) permeability.

There are numerous reports linking redox biology with maintenance of the intestinal epithelium, primarily through the NADPH oxidases (NOX) and generation of H_2_O_2_ [128]. It was demonstrated that NOX1 is highly expressed by the proliferation of colonic stem cells, which promote self-renewal. NOX1 generating H_2_O_2_ can oxidize cysteines in endothelial grown factor receptor (EGFR) to potentiate its activation and stimulate proliferation [129]. This pathway also could activate the Nrf2/NF-κB axis for adaptation or cytotoxicity depending on H_2_O_2_ concentration generated by the NOX enzymes [95,130]. This feedback loop supported prolonged proliferation of colon stem cells by the presence of bacteria in the colonic lumen which by microbial and other sources of TLR ligands activate TLR4 which simulates NOX1 and dual oxidase 2 (DUOX2) expressions. At higher activation epithelial TLR4 signaling activates NOX1 and DUOX2 to generate high concentration of H_2_O_2_ and induces microbiota driven tumorigenesis [131]. We believe that daily diet of polyphenols and polyphenol metabolites at the surface of the colon epithelium generating low concentration of H_2_O_2_ induce epithelial oxidative eustress [2,132], (see Figure 1).

Food sources of oxidants such as a high-fat diet (HFD) and oxidized oils in the presence of red meat containing iron catalyzers induce lipid peroxidation and the generation of reactive carbonyls and advanced lipid oxidation/glycation end products (ALEs/AGEs). These products activate receptor advanced glycation end products (RAGE) which activate NADPH oxidase (NOX) generating hydrogen peroxide (H_2_O_2_). An HFD induces the growth of microbiota, and the generation of lipopolysaccharides (LPS) activates the signaling nuclear factor kB (NF-κB) and NOX, both highly increase production of H_2_O_2_ which also activates NF-κB. Other lipid oxidizing factors such as oxy-cholesterol activate EGFR (endothelial growth factor receptor), TLR4 (toll-like receptors), and PI3K/Akt (phosphoinositide-3-kinase/protein kinase B); ERK (extracellular signal-regulated kinase) activates NF-κB generated inflammatory cytokines and protein receptors EGFR, NOX, RALEs, but decreasing generation of TJs (tight junctions) such as AJ (adherence junction), GJ (gap junction) and desmosome proteins increasing GI permeability. Polyphenol antioxidants act through good timing during the meal in the aerobic stomach to inhibit lipid peroxidation and generation of reactive carbonyls ALEs and AGEs. Generation of H_2_O_2_ at low levels activates the Nrf2 signaling factor, generating protective proteins and enzymes, increasing all the tight junction proteins and preventing GI permeability. Polyphenol by keeping a good microbiota in the colon prevent generation of LPS, activation of TLR4 and formation of inflammatory cytokines and in general cellular eustress.

## 5. Cardiovascular System

**Figure 2:**
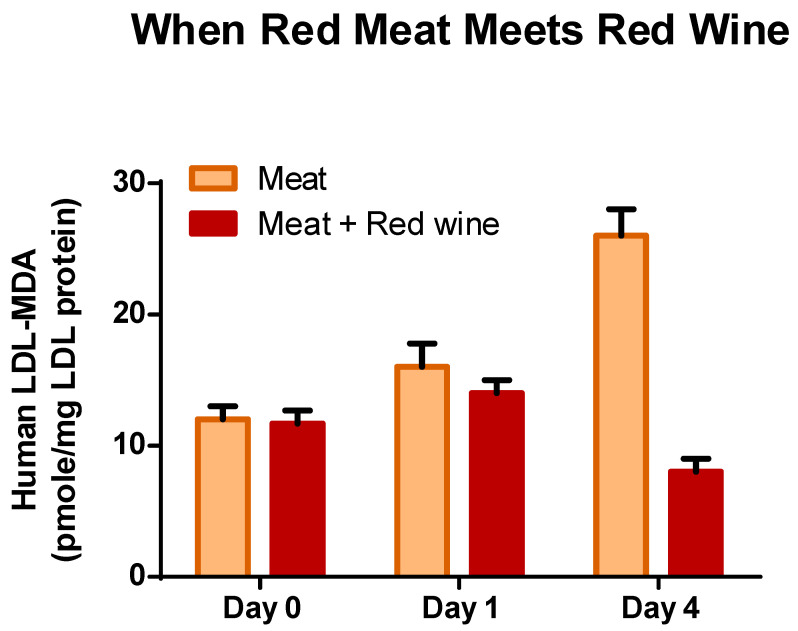
The timing effects of 4 days supplementation of red-wine polyphenols (527 mg) during a meal containing turkey red-meat (250 g) on plasma LDL-MDA modification in human. Reproduced and changed from Gorelik et al., 2013 [141].

Polyphenols compounds in foods have attracted a great deal of interest since the 1990s due to growing evidence of their potential beneficial effect on human health. Our early report that beverages such as red-wine contain a high concentration of polyphenol substances could inhibit LDL oxidation ex-vivo stimulated huge interest in this field [133,134,135,136]. The LDL oxidation hypothesis of atherosclerosis triggered extensive investigation on the role of antioxidants including polyphenols against the onset and development of oxidized-LDL induced atherosclerosis [134,137,138]. It was assume that polyphenols containing foods and beverages exert potent antioxidant effects in vitro, but were not as efficient in vivo primarily due to low bioavailability and high-metabolism which affect their effectiveness [139]. However, epidemiological studies, experimental investigations and clinical studies support the concept that polyphenol-rich foods can improve the vascular function in humans. Clinical studies with human volunteers demonstrated that simultaneous consumption of red meat with a food rich in polyphenols (red wine or black coffee) prevents both lipid peroxidation and generation of MDA in the stomach and absorption of MDA-derivative to increase plasma MDA and modified MDA-LDL [26,41,140] (see Figure 2). Reactive carbonyls play critical roles in the pathogenesis of atherosclerosis [141,142], but also in many other chronic diseases such as diabetes, kidney diseases, retinopathy, neuropathy and cancer [143,144]. The pathological effects of reactive carbonyls are related to their ability to modify reactive proteins or DNA by cross-linking, protein oligomerization, immune-responses or to bind to RAGE (receptor for AGEs-advanced glycation end-products), activating the NADPH oxidase and generating reactive oxygen species [143,145,146,147]. Such interaction with proteins and receptors could promote cellular oxidative stress, inflammatory mediators, and chronic diseases and cancer [143]. Modified-LDL or MDA-LDL is recognized as a key factor in the initiation and acceleration of atherosclerosis [148], but also as a marker of coronary artery disease severity [149] and others cardio-vascular diseases [150,151].

The consumption of red meat during a meal, as a model for increasing lipid oxidation end-products (ALEs) in the blood system in human, was adopted by other investigators for preventing it by consuming with the meal foods rich in polyphenols [24,43,46,47]. Many other in vitro studies demonstrated the excellent antioxidant activity of polyphenols or foods rich in polyphenols on lipid peroxidation in stomach medium or GIT [28,152,153]. The PREDIMED group suggested that the health benefit of the Mediterranean diet should be attributed to the high consumption of foods containing polyphenols [154,155]. We suggest that the main benefit of consuming plant polyphenols and other redox compounds in the human diet, as an integral part of the meal, arises mainly from the ability to prevent in the stomach lipid peroxidation, co-oxidation of vitamins, generation of reactive aldehydes and other cytotoxic ALEs. Due to this action the polyphenols decrease the generation and absorption of reactive carbonyls and other cytotoxic compounds into the blood system and increase the bioavailability of vital vitamins. Both, these factors act synergistically for improving human health. Some authors believe in a paradigm shift in antioxidant research, from antioxidant capacity to anti-inflammatory effects in gut health [16]. However, the truth is that antioxidant capacity, anti-inflammatory and many more effects should be attributed to antioxidants, and especially to polyphenols, for human health.

### 5.1. Polyphenols Action in the Cardiovascular System

There is much evidence to support a potential beneficial action of polyphenols consumption on cardiovascular health [156]. The ultrastructure of terminal mammalian arterioles is composed by endothelial cells which have the same height and reach at the thinnest area a diameter of ~0.15 µ [157]. This arteriole ultrastructure permitted the blood system to be excellently connected with our organs for a perfect transfer of nutritional elements and exchange of gas molecules especially O_2_, CO_2_, H_2_S and NO. This ultrastructure also permitted the polyphenols to be in high interaction with the endothelial cell membranes, most probably by hydrophobic or hydrogen bonding of polyphenol hydroxyls to protein or phospholipid amine groups.

Due to the poor absorption and extensive metabolism in the enterocyte polyphenols undergo extensive metabolic transformation but still retain significant redox capabilities [158,159]. The bioavailability of specific polyphenol molecule in the blood system could attain a concentration of ~ 1µM, but when in combination with other polyphenols, they could reach a higher concentration. It is most likely, that polyphenols may act in-vivo via the pro-oxidative effects following reactions which generate H_2_O_2_, semiquinones and quinones [160]. Once polyphenols form the hydroquinone cation radical or the semiquinone (Reaction (6)), oxygen molecules react with the radicals at near diffusion rate generating H_2_O_2_ and quinones (Reactions (2)–(4)). These reactions initiate the auto-oxidation of polyphenols by which in the presence of reduced phenols the quinones are disproportionated to two semiquinones (Reaction (5)), which interact with oxygen to form more O_2_^•−^ and H_2_O_2._ The auto-oxidation of polyphenols in the blood system could be enhanced by other reducing compounds such as ascorbic acid which reduce quinone to semiquinone and by these to attain higher concentration of H_2_O_2_. At the extracellular endothelial cell membranes area SOD_3_ (the extracellular enzyme) may transform O_2_^•−^ to more H_2_O_2_. Hydrogen peroxide, generated at extracellular side, will diffuse across membranes through aquaporins, known as peroxiporins [161], into endothelial cells affecting redox-cellular responses via activation of signaling factors [162,163,164]. After diffusion inside the cells and near the membrane, a very low concentration of 1–10 nM H_2_O_2_ will affect cell proliferation and angiogenesis. At a concentration of 10–100 nM will affect adaptation to stress responses, but at higher a concentration of 1 to 10 µM H_2_O_2_ will induce inflammation and cell death [132]. All those processes were demonstrated in vitro and in vivo to be affected by various polyphenols [2].

### 5.2. Proliferation and Angiogenesis

The ability of apple extracts to inhibit proliferation of tumor cells in vitro was attributed to polyphenols [165]. We and others demonstrate that polyphenols at very low concentration, in cell culture, generate low concentration of H_2_O_2_ which increases cell proliferation and wound repair. However, at high-concentration due to high-generation of H_2_O_2_ they inhibit proliferation, angiogenesis, wound repair and decrease cell survival [10,11,166,167].

### 5.3. Protection, Adaptation, and Cell Surviving

Preconditioning of cells with low concentration of H_2_O_2_ (10 µM) protects the same cells from subsequent 6 mM of H_2_O_2_-induced cytotoxicity [168]. H_2_O_2_-preconditioning of cells was found to modulate phase-II-enzymes through mitogen-activated protein kinase (MAPK), and PI3K/Akt kinase activation [169,170], by these preventing cytotoxicity affected by many toxins. H_2_O_2_ at low concentration generated from ternary butylated quinone (t-BHQ), resveratrol and curcumin directly activate Nrf2 and the phase-II-enzymes [2,160]. Many other polyphenols were found in-vitro to prevent cell cytotoxicity, most probably via generation of H_2_O_2_. Baicalein protects cardiomyocytes or neuroblastoma cells from hypoxia reoxygenation and H_2_O_2_-induced cytotoxicity, respectively [171,172]. Hydroxytyrosol, the main polyphenol in olive oil and leaves, inhibits H_2_O_2_–induced cell injury in vascular endothelial cells by activation of kinases and expression of Nrf2 which induce heme-oxidase 1 (HO1) and up-regulate catalase expression through the AMP-activated protein/Forkhead box protein O3 (AMPK-FoxO3a) pathway [167,173]. Polyphenols act for protection and adaptation not only in in vitro cell culture but also in vivo with animals and humans. An increase in endothelial nitric oxide syntase (eNOS) expression in aorta was observed in in vivo studies by the intake of wine polyphenols or resveratrol in rats [174,175]. Epidemiological studies have indicated that a regular intake of polyphenol-rich diets such as fruits, vegetables, red wine, tea or cocoa are associated with a reduced risk of cardiovascular diseases [176,177]. The dietary intake of polyphenols is highly variable and no food contains only a single class of polyphenols. Due to catabolic and metabolic reactions of the parent compounds, the absorbed constituents in the blood vessels, at a low micromolar concentration, retain in part the reducing potential and the synergism between polyphenols (reaction # (6)) to generate H_2_O_2_, in the presence of oxygen, targeting the endothelium blood system. As is known, like H_2_O_2_ [178,179,180,181] polyphenols through intercellular and intracellular generation of H_2_O_2_ [160], may affect endothelial formation of NO and endothelial-dependent hyperpolarization (EDH), both induce vasorelaxation. In the blood system the endothelial cells are mostly affected by the action of polyphenols due to higher physical interaction. Several studies demonstrated that grape/wine polyphenols due to a pro-oxidant effect generate H_2_O_2_ which affects redox-cysteine sensitive up-regulation of eNOS via activation of PI3-kinase/Akt, Protein-38 (p38), MAPK, c-Jun N-terminal kinase (JNK), and inactivation of FoxO1 and FoxO3a [182,183]. These effects are induced through activation of PI3-kinase/Akt/eNOS pathway which generates NO [183].

## 6. Polyphenols and Diabetes

### 6.1. Beta Cells

Many studies have revealed that high glucose and free fatty acids induced glucolipotoxicity in islet cells and increased intracellular ROS and apoptosis. High H_2_O_2_ can impair ß-cells function via decreased levels of two transcription factors pancreatic duodenum homeobox-1 (Pdx-1) and MafA which affect insulin gene expression, insulin content and secretion [184]. On a chronic basis, it seems that low oxidative stress leads to induction of the antioxidant enzymes due to activation of Nrf2 to blunt ROS signaling, glucose stimulating insulin secretion (GSIS), and deterioration of islet function [185]. The excessive production and accumulation of ROS are due to hyperactivity of NADPH oxidases. The NADPH oxidase 4 selective inhibitor GLX7013114 counteracts human islet cell death in vitro [186]. There is much evidence that polyphenols preserve ß-cells integrity and insulin secretion against oxidative damage. Quercetin potentiates insulin secretion and protects INS-1 pancreatic ß-cells against ROS via the ERK1/2 pathway [187]. Curcumin protects ß-cells from glucolipotoxicity in vitro and in vivo streptozotocin induced T2D (type 2 diabetic) rats [188]. Morin protects pancreatic ß-cells against ROS induced DNA damage by activating Nrf2 signaling pathway [189]. The importance of Nrf2/antioxidant signaling pathway was also found to mediate ß-cells self-repair after damage by high-fat diet-induced oxidative stress. It was found that acute hyperglycemia in Zucker diabetic fatty rats fed a high-fat diet generated oxidative stress and ß-cells structural damage; however, when returned to low fat diets ß-cells repair themselves via a mechanism depending on Nrf2 activation and generation of intracellular antioxidant proteins [190]. In addition, dipeptidyl peptidase-IV inhibitor which increases insulin secretion has drawn wide attention as a new treatment strategy for T2 diabetes. Several polyphenols were found to inhibit the enzyme dipeptidyl peptidase-IV and allowed two critical hormones in the GIT, glucose dependent insulinotropic polypeptide and glucagon like peptide-1 to stimulate insulin secretion and lower blood glucose [191].

### 6.2. Diabetes, Liver and Hepatic Gluconeogenesis

In hepatocytes, elevated free fatty-acids (FFA) levels lead to ectopic fat deposition (storage of triglycerides in tissue other than adipose tissues), which consequently inhibits insulin receptor substrate (IRS2)-associated Akt/PI3K cascade activation and glucose transporter 2 (Glut2) expression, reducing insulin-stimulated glucose uptake (insulin resistance) [192]. Fat-deposition induced inhibition of Akt/PI3K decreases the phosphorylation of FOXO1, which, as a result, activates the transcription of glucose-6-phosphatase (G6Pase) and phosphoenolpyruvate carboxykinase (PEPCK), the rate-limiting enzymes for gluconeogenesis [193]. The resulting increased hepatic glucose production leads to hyperglycaemia and the development of T2DM [194].

Polyphenols from cinnamon extracts (CE) have been reported to improve insulin sensitivity and glucose homeostasis by regulating hepatic enzymes activities, attributed to its phytochemical composition such as cinnamic acid, cinnamaldehyde and proanthocyanidins. Supplementation of rat hepatoma cells (H4IIE) with (1–25 µg/mL) CE was demonstrated to inhibit hepatic glucose production by downregulating the expression of PEPCK and G6Pase concomitantly decreasing blood glucose levels. Such insulin-like and glucose-lowering effects of CE may help to ameliorate T2D conditions [195]. Morin has been reported to enhance the insulin action in cultured cells promoting few metabolic responses. Paoli et al. found that Morin increases the phosphorylation of the insulin receptor and Akt in a dose-dependent manner and inhibited gluconeogenesis and enhanced glycogen synthesis as demonstrated using liver cells, HepG2 [196]. Moreover, they demonstrated the activation of FOXO1 signaling cascade by Morin thereby inhibits the gluconeogenesis pathway. Epigallocathechin-3-galate (EGCG), in addition to its potent insulin secretion abilities, also inhibits glucose production in hepatocytes. Incubation of H4IIE cells with EGCG (5–25 µM) was shown to suppress PEPCK and G6Pase genes via PI3K activation in a dose-dependent manner, resulting in reduced hepatic glucose output. It was also shown in the same study that treatment of H4IIE cells with EGCG promoted tyrosine phosphorylation of insulin signaling proteins such as insulin receptor (IR-β), IRS-1 and insulin grow factor 1 receptor (IGF-1R) through PI3K/Akt activation, owing to its insulin-mimetic properties [197]. Several studies concurred that EGCG suppression of hepatic gluconeogenesis was dependent on initial production of ROS, a known activator of Ca^2+^/calmodulin-dependent protein kinase kinase (CaMKK) [198]. Epigallocathechin-3-galate at sub-micromolar concentration suppresses hepatic gluconeogenesis through generation of H_2_O_2_ (which was prevented by PEG-catalase and MnTMPyP) and activation of AMP-activated protein kinase (AMPK) mediated by CaMKK [199]. Very similar to H_2_O_2_, polyphenols activate formation of NO through Ca/Calmodulin [200], activate estrogen receptor, CaMKK, AMPK and Sirt1, most probably by generation of H_2_O_2_ [200,201].

### 6.3. Diabetes, Cardiovascular, Muscle Cells, Adipocyte, and Kidney

Glucose homeostasis in the organism is affected by redox regulated mechanism. ROS signaling contributes for many physiological functions and also for processes of hormone synthesis, insulin secretion, and insulin sensitivity. Dysfunction in ROS signaling includes formation of excessive amounts of ROS, appearance of ROS at non-physiological subcellular sites or in cell types that normally do not form relevant amounts of ROS, or shifting from a physiological to a non-physiological type of ROS, e.g., from hydrogen peroxide to superoxide [202]. To understand the effects of polyphenols on preventing and ameliorating T2-diabetes and the complexity of ROS pathobiology we need a short introduction.

In peripheral insulin-sensitive tissues such as skeletal muscle, fat cells, kidney, neuron and liver cells, insulin controls the switch from lipolysis/fatty oxidation during fasting to lipid storage/glucose oxidation following feeding. Binding of insulin to the insulin receptor (IR) phosphorylates substrate proteins, IRS1 and IRS2, activating phosphatidylinositol 3-kinase (PI3K)/Akt (protein kinase B) signaling, which leads to the translocation and activation of glucose transporters (mainly GLUT4 in muscles and fat cells) and subsequent glucose uptake [203]. ROS, at low concentration, comes into play in insulin signaling through activation of PI3K and alternative protein kinase C (PKC) activation to increase NOX4 activity, forming H_2_O_2_. H_2_O_2_ augments insulin-IR-PI3K signaling twofold by inhibiting protein tyrosine phosphatase 1B (PTP1B) and the phosphatase and tensin homologue, PTEN, which dephosphorylates IR and downregulates PI3K signaling and by activating MAP kinase phosphatase-1, which dephosphorylates IRS1 [200,203]. Further increased ROS production is associated with peripheral insulin resistance, a main feature of T2DM. In early stages of T2DM, NOX4 causes fat and liver cell inflammation, apoptosis and fibrosis [201,204].

In blood vessels, ROS have been suggested to cause hypertension, atherosclerosis, and a pro-thrombotic stage, either directly or by interfering with protective NO [58,160]. Surprisingly, however, this does not account for all ROS; NOX4-derived H_2_O_2_, at low concentration, is anti-atherosclerotic by reducing fibrosis and proliferation of smooth muscle cells [205]. These examples show the complexity of ROS pathobiology with different sources/types of ROS having qualitatively opposing effects, making precise targeting of the most disease-relevant isoform pertinent for any chronic therapy in T2DM. Superoxide appears to be the most disease-relevant type of ROS. It can decrease NO bio-phase levels by direct chemical scavenging, leading to intermediate peroxynitrite, protein tyrosine nitration, reducing endothelial insulin receptor expression, and inhibiting phosphatidylinositol 3-kinase (PI3K)-Akt-NOS3 signaling in the endothelium [183]. In addition, superoxide uncouples NOS3, which decreases NO production and simultaneously increases superoxide production from uncoupled NOS3 (i.e., an example of ROS-upgrading). Finally, superoxide and/or peroxynitrite can damage the NO receptor, sGC leading to a collectively threefold interruption of NO-cGMP signaling by: (i) scavenging of NO, (ii) uncoupling NOS3, and (iii) damaging the NO receptor sGC [202].

There is much evidence to support a potential beneficial action of polyphenols consumption on type 2 diabetes mellitus [206,207]. It is well-known that low concentrations of cell exogenous H_2_O_2_ (50–100 nM) inhibits PP2A and other PTPs (protein tyrosine phosphatases), thereby increasing the level of protein phosphorylation [2,208]. It seems that nano-molar concentration of caffeic acid/H_2_O_2_, which partially inhibits PP2A, increases phosphorylation and nuclear Nrf2, and decreases nuclear p65 (protein 65); thus, in this way, prevents deregulations of the cells by high glucose. Nano-molar concentration of caffeic-acid attenuates glucose-induced endothelial cell dysfunction by affecting NF-κB and Nrf2 pathways [209]. Nrf2-mediated inhibition of the inflammatory cytokine gene expression is ARE-independent. Nrf2 specifically inhibits the inflammation-induced transcription mediated by NF-κB. This notion coincides with the fact that Nrf2 also binds to the Interleukin-6 and Interleukin-1b (IL-6 and IL-1b) genes’ loci and inhibits their transcription [203]. In general, at the same time, Nrf2 upregulates expression of genes coding antioxidant proteins and downregulates target genes that encode inflammatory cytokines, and in this way, eliminates ROS and subsequently contributes to the anti-inflammation process [203]. In mice fed a high-fat diet, Daveri et al. [210] have shown that polyphenols modulate inflammation and alter redox signaling, improving insulin resistance. Several studies in vivo on tea polyphenols, and especially EGCG, via dampening of PTP1B (protein tyrosine phosphatase 1B) and other PTPs acting as key regulators of tyrosine phosphorylation-dependent signaling accelerate glucose uptake and evoke the IRS-1/Akt/GLUT2 signaling pathway in HepG2 cells and mice liver have been conducted [211]. By inhibition of PTP1B, EGCG stimulates nuclear translocation of Nrf2 after provoking the PI3K/Atk signaling pathway, and thus modulates the expressions of antioxidant enzymes such as HO-1 and NQO1 [211], most probably by activation of Nrf2 transcription. Furthermore, EGCG supplemented to mice significantly ameliorated high-fat high-fructose diet (HFFD)-triggered insulin resistance and postprandial oxidative stress, cognitive defects by upregulating the IRS-1 (insulin-receptor substrate 1)/Akt, Keap/Nrf2 and ERK/BDNF/CREB (brain-derived neurotrophic factor/c-AMP-response element binding protein) transcription pathways [211,212]. EGCG also ameliorates in mice the metabolic syndrome derived from HFFD, by increasing brown adipose tissue (BAT) energy expenditure and preventing adipocyte hypertrophy and fat accumulation [212,213]. BAT is a major regulator of thermogenesis in mammals. A high fat diet (HFD) was found to promote the growth of flavonoid-metabolizing bacteria, which in turn decreases the number of bioavailable flavonoids which are important to ameliorate post-dieting obesity. Weight-adjusted energy expenditure was markedly reduced in weight-cycling mice, but was normalized upon flavonoid administration [122,123,213]. The research shown that polyphenols administration elevated significantly the thermogenic factor uncoupling protein-1 (UCP1) transcript levels in BAT of mice fed the HFD [124,125,126]. It seems that this is an important pathway by which flavonoids may affect overweight and prevent T2 diabetes. However, the activation of UCP1 is also induced by H_2_O_2_, generated by auto-oxidation of phenols [127,214]. Interesting results were published on the possible therapeutic potential of aspirin beyond its ability to inhibit cyclooxygenase pathways. The researchers found that aspirin and salicylic acid are partially metabolized to di-hydroxy-benzoic acid (polyphenol) generating H_2_O_2_ which acts as inducers of Sirt1 and other downstream targets of Sirt1, PGC-1α and AMPK [127,214].

Polyphenols seem to ameliorate these deleterious pathway by generating low concentration of H_2_O_2_ in arterioles, which interacts exogenously with endothelial cells, penetrates into cells, and inhibits phosphatases [215]; thus, increasing phosphorylation of several anti-inflammatory signaling factors and especially the Nrf2 target genes. These activities are not relevant to the blood system alone, but because the blood system are present in all organs, it seems to beneficially affect all of them. It seems that the involvement of polyphenols as a pro-drug generating low concentration of H_2_O_2_ acts beneficially in several more systems.

Chronic kidney disease (CKD) is characterized by the feature of accelerated ageing, increases levels of cellular senescence, vascular calcification, osteoporosis, sarcopenia, frailty and depression. Diets that are potentially beneficial in CKD include a vegetarian diet or a low protein diet with a reduced intake of red meat. These diets have been associated with a reduction in uremic toxins. Red meat during heat cooking and after postprandial stomach digestion generates by lipid peroxidation a high level of reactive carbonyls [26] compounds which reach and affect kidney dysfunction [120,143]. The treatments for early stage of CKD are to slow the progression of the disease and to control uremic complications, such as inflammation, high blood pressure, insulin resistance and metabolic acidosis. Studies have reported that plant-based diets rich in fruits, vegetables, tea, cocoa, coffee, and all whole grain cereals could potentially slow down the progression of CKD [216]. All those foods are rich in polyphenols [48].

## 7. Polyphenols Neurons, Brain Function and Ageing

The central nervous system (CNS) is particularly sensitive to oxygen level and reactive oxygen species, the deregulation of the redox balance, oxidative distress, is strongly linked to neurodegeneration, such as Alzheimer’s, Parkinson’s, and Huntington’s diseases, and Amyotrophic Lateral Sclerosis [217,218,219,220]. Insult to the CNS cause different NOX activation and generation of H_2_O_2_. Generally, at low level, this response is protective by clearing debris and supporting neuronal survival. However, at a high concentration H_2_O_2_ causes neuronal auto-toxicity and breakdown of the cell membrane barrier [221].

All humans develop ageing but only some die at a very late age. Apart from genetic factors it seems that diet affects the genesis of ageing very much. Diet is at the cross-roads of many human chronic diseases affecting ageing. The free radical generation mechanisms provided a basis for the free radical theory of ageing [222]. Damaging roles of oxidants are well known to affect ageing [223]. Cellular senescence contains cells that are metabolically active but not contribute to physiological functions and may persist in tissues and organs for many years [224]. In a young tissue or organ less than 1% of the cells are senescent, but this proportion rises to more than 5% with biological age [225]. Senescence cells are characterized with senescence-associated secretory phenotype (SASP) which mediates a toxic pro-inflammatory environment and promote cellular senescence in distal tissues [226].

Cardiovascular diseases, T2-diabetes and uremic vascular calcification are characterized by increased cellular senescence and could be treated by senolytic drugs such as Dasatinab [226,227,228]. Polyphenols such as quercetin, resveratrol, curcumin, rutin, catechin, berberine, fisetin or proanthocyanidin were found to act as senolytic compounds by activating the cellular Nrf2 axis [2,229,230,231]. Polyphenols mediate anti-ageing effects also through activation of Sirt1 which down regulates senescence related proteins and pro-inflammatory cytokines and vascular ageing [232,233,234]. Many of the active polyphenol derivatives, absorbed in the blood system, are alkyl catechols derived from the colonic microbial processing of polyphenols that are present in fruits, vegetables, red wine, tea, coffee or cacao which are found in Mediterranean diets and are missing in the Western diet [48,235,236]. Those alkyl catechols could by auto-oxidation in the surface of blood endothelium to generate electrophiles and H_2_O_2_ and after penetration into cells to activate the Nrf2 axis [2], ameliorating cellular senescence in distal tissues, affecting ageing [217,237]. Studies with Caenoehabditis elegans found that early-life exposure to ROS or chlorogenic acid impacts stress resistance later to life extending lifespan [238,239]. Hydrogen-peroxide was found to cause oxidant-sensitive epigenetic changes that increased stress resistance and lifespan [217,238]. Redox regulation of ageing by H_2_O_2_ or polyphenols seems to be affected pleiotropically [2,240].

Polyphenols were found to ameliorate age-related cognitive decline and neurodegenerative diseases. The beneficial effects of polyphenols on brain function seem to act by modulating signaling pathways, promoting cerebrovascular blood flow, controlling synaptic plasticity, reducing neuro-inflammation, stimulating new nerve cell growth, and attenuating extracellular accumulation of pathological proteins. There are several studies on bioavailability of polyphenols in brain tissues founding some transfer, but further work is necessary to confirm that polyphenols can diffuse in the brain and directly modulate brain function [241]. Hesperitin was founded to affect Akt and ERK1/2 activation status in cortical neurons [242]. In mice, hesperidin was founded to attenuate learning and memory deficiency in APP/PSI mice (β-amyloid precursor protein/presenilin1) through activation of Akt/Nrf2 signaling and inversely affected by reactive carbonyls through receptors of advanced glycation end-product (RAGE), which activates the NOX1/H_2_O_2_/NF-κB pathway [243]. Dietary supplementation with tBHQ (tert-butylhydroquinone), an Nrf2 activator, confers neuroprotection against apoptosis in amyloid β-injected rats [244]. T-BHQ was found to confer neuroprotection in vivo in several more studies [245,246,247]. Curcumin and tannic acid provide neuroprotection in in vivo models of traumatic brain injury and cerebral ischemia-reperfusion via Akt and mTOR (mammalian target of rapamycin) and the Peroxisome proliferator-activated-receptor-gamma-coactivator-1alpha (PGC-1α)/Nrf2-ARE (antioxidant response element) signaling pathways [248,249]. Resveratrol was also found to confer neuroprotection in mice against aging-related deficits through an ERK1/2-dependent mechanism [250]. Tea polyphenols or EGCG supplemented to mice significantly ameliorated postprandial oxidative stress, cognitive defects by upregulating the IRS-1/Akt/Keap/Nrf2 and ERK/BDNF/CREB (cAMP-response element binding protein) transcription pathways [251]. It seems that EGCG potentiates the brain-derived neurotrophic factor proteins (BDNF) action by generating of H_2_O_2_ since this effect was abolished by catalase [252]. All above mentions are about polyphenols which differ in molecular configuration, classes and molecular size but all generate H_2_O_2_ by auto-oxidation [160]. The protective effects of polyphenols seem to be mediated by an indirect mechanism, affected by an exogenous low concentration of H_2_O_2_ flow, and generated at the level of the blood–brain barrier (BBB) cells. Polyphenols such as t-BHQ, curcumin and resveratrol activate the Nrf2 pathway in astrocytes by exogenous H_2_O_2_ [160]. The importance of exogenous H_2_O_2_ generation delivering redox signaling for healing of axons were published [253,254]. Hervera et al. [253] identified a new physiological role for H_2_O_2_ in the brain in which it acts as trans-cellular signaling, established by exosome-mediated NOX transfer as a mechanism for this pathway. The exosome is generated from macrophages, recruited, and attracted to the localized tissue injury, which produces H_2_O_2_, and helps to transfer the effect at a distance. The generation of H_2_O_2_ at low concentration, from the exosome, causes oxidation-induced inactivation of PTEN (phosphatase and tensin homolog); which enables activation of the PI3K/Akt signaling pathway, and leads to adaptation and enhanced survival for cells and beneficial effects for the brain. In several more experiments, polyphenols ameliorated the postprandial oxidative stress, induced by cell culture supplemented with glucose-amines or in mice with the high-fat high-fructose diet (HFFD), both generating AGEs in the model system or organism [211,212]. The experiment in mice [243] by which polyphenols attenuate learning and memory through activation of Akt/Nrf2 signaling and inhibition of the RAGE/NF-κB pathway, integrates two main factors affected by polyphenols; (a) the antioxidant action mainly induced in GIT which inhibits the generation of cytotoxic carbonyls (AGEs/ALEs), preventing the activation of the RAGE/NOX/NF-κB distress pathway; (b) in blood system polyphenols by generating H_2_O_2,_ at nM concentration, activates in endothelial cells the PI3K/Akt/Nrf2 signaling eustress axis for cell adaptation and protection [2].

## 8. Conclusions

**Figure 3:**
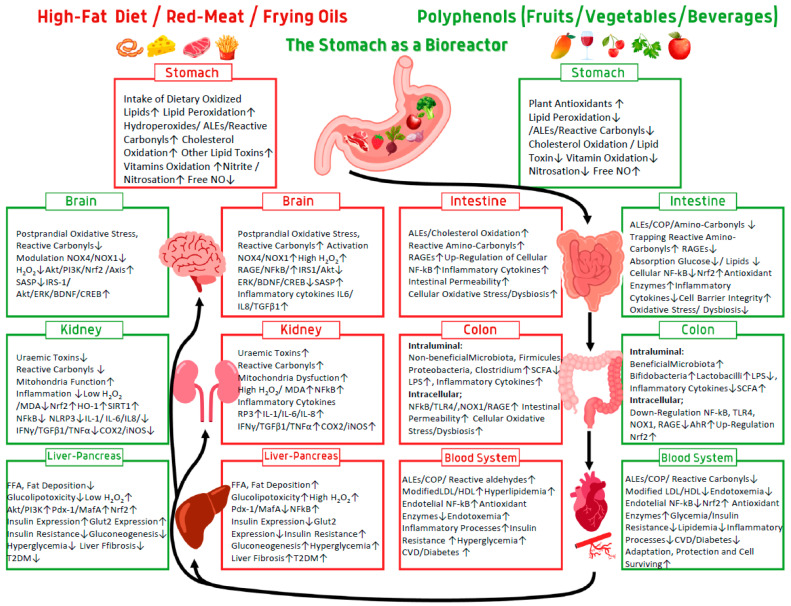
Polyphenols mode of action as preventive medicine. The stomach acts as a bioreactor, generating with some foods and especially muscle-foods free radicals and toxic compounds, inducing pathologic pathways in human metabolism. These pathologic pathways could be prevented during the meal by food antioxidants and mostly by polyphenols. Polyphenols at the stomach and GIT level synergistically affect the system as antioxidants and inhibitors of dietary catalyzers of lipid oxidation and generators of reactive aldehydes, AGEs/ALEs. Polyphenols act also as scavengers of free radicals, trapping reactive carbonyls, modulating digestive enzyme activity, increasing the growth of beneficial gut microbiota and inducing eustress cellular signaling. At endothelial blood level, at nM low concentration, polyphenols act as generators of electrophiles and low concentration of H_2_O_2_, acting mostly as cellular signaling, activating the PI3K/Akt-mediated Nrf2/eNOS pathways, inhibiting the transcription of NF-κB and inducing the cells, organs and organism for eustress, adaptation and surviving. Keeping timing of the redox homeostasis in stomach medium, GIT and cardiovascular system during the meal affect also other organs such as liver, pancreas, kidney and brain, seems to be the golden means of healthy living. All these activities applying at the right time synergistically provide polyphenols to act in humans as preventive medicine.

We consider that the stomach is an important organ in our body which acts as a bioreactor generating with some foods and especially muscle-foods, free radicals, and toxic compounds, inducing pathologic pathways in human metabolism. These pathologic pathways could be prevented during the meal by food antioxidants and mostly by polyphenols. Polyphenols at the stomach and GIT level affect synergistically the gut system as antioxidants and inhibitors of dietary catalyzers of lipid oxidation and generators of reactive aldehydes, AGEs/ALEs. These act as scavengers of free radicals, trapping reactive carbonyls, modulating digestive enzyme activity, increasing the growth of beneficial gut microbiota, and inducing eustress cellular signaling (Figure 3). At the endothelial blood level, at nM low concentration, they act as generators of electrophiles and low a concentration of H_2_O_2_, acting mostly as cellular signaling, activating the PI3K/Akt-mediated Nrf2/eNOS pathways, inhibiting the transcription of NF-κB and inducing the cells, organs and organism for eustress, adaptation and survival. Many large scale human intervention studies on the effect of dietary antioxidant supplements did not demonstrate preventative or therapeutic effects of the antioxidants [255]. I suggest that these null effects of the antioxidants were achieved mostly because of inappropriate timing and in dosage supplementation. Keeping timing of the redox homeostasis in stomach medium, GIT and cardiovascular system during the meal seems to be the golden mean of healthy living. All these activities applied at the right time and concentration synergistically integrate polyphenols to act in humans as a preventive medicine.

## Figures and Tables

**Table 1:** Food polyphenols in fruits, vegetables, and beverages.

Food (var.)	Polyphenols mg/100 g FW	IC_100%_/g FW ^a^	rPOSI ^b^
Blackberries	667 ± 4.5	16	625
Quince (Portugal)	460 ± 6.3	18	568
Blueberries	310 ± 6.7	42	240
Pomegranate (Wonderful)	145 ± 3.5	72	138
Pear (Spadona)	100 ± 3.8	104	96
Strawberries (Tamar)	190 ± 4.7	137	74
Purple Grapes (Red Globe)	150 ± 1.5	160	63
Banana (Ziv)	114 ± 4.2	212	47
Peach (White Lady)	168 ± 6.2	288	35
Spinach (Winter)	105 ± 2.0	105	96
Broccoli (Monaco)	85 ± 3.5	161	62
Onion (Yellow)	80 ± 3.2	402	25
Red Beet	196 ± 6.0	370	27
Eggplant (Black)	171 ± 2.5	460	22
Red Wine (Petite Sirah)	237 ± 5.1	168	60
Black Coffee (Turkish-ground)	225 ± 6.3	169	59
Tea (Green)	125 ± 2.3	416	24
Tea (Black)	113 ± 5.1	657	15
Coffee (Freeze-dried)	200 ± 5.3	752	13

^a^ Food amount in grams (FW) for 100% inhibition of 200 g turkey red-meat lipid peroxidation in stomach medium = rPOSI 100. ^b^ rPOSI (redox Postprandial Oxidative Stress Index) = The inhibition index of 100 g of the food. Beverages presented in (mL). Reproduced and changed from Kanner et al., 2017 [48].

**Table 2:** Mediterranean Greek salad containing a collection of polyphenols calculated by rPOSI for neutralize ePOSI 100.

Foods-100 g	Polyphenols (mg)/100 g	IC_100%_/g FW	rPOSI
Tomato	29	625	16
Cucumber	0	0	0
Red-Pepper	47	193	53
Green-Cabbage	24	240	42
Onion (Purple)	120	280	36
Olive (Manzanillo 25 g)	80	54	45
**Total/525 g salad**	**300**		**192**
**A Salad portion of 274 g**	**156**		**100**

Turkey red-meat lipid peroxidation by 200 g in stomach medium = ePOSI 100. rPOSI (redox postprandial oxidative stress index) = The inhibition index of 100 g of the food. ePOSI 100 should be reduced by rPOSI 100 to POSI 0. Reproduced and changed from Kanner et al., 2017 [48].

## Data Availability

Not applicable.

## Abbreviations

Malondialdehyde (MDA), glucose transporter (GLUT2), nuclear factor 2 (Nrf2) interleukin (IL)-8, IL-1β, tumor necrosing factor alpha (TNF-α), heme oxygenase 1 (HO-1), superoxide dismutases (SODs), and glutathione peroxidase (GPx), toll-like receptors (TLR), short chain fatty acids (SCFA), tight junctions (TJ), adherence junction (AJ), gap junction (GJ), phosphoinositide-3-kinase/protein kinase B (PI3K/Akt), protein kinase C (PKC),uncoupling protein-1 (UCP1), NADPH oxidases (NOX), aryl-hydrocarbon-receptor (Ahr), pancreas/duodenum homeobox protein 1 (PDX1), musculoaponeurotic fibrosarcoma oncogene homolog A (MAFA), low density lipoprotein (LDL), high density lipoprotein (HDL), cardiovascular diseases (CVD), type-2 diabetes (T2-D), extracellular signal kinase (CRK), interleukin 1/6/8 (IL-1/6/8),brain-derived neurotrophic factor (BDNF), cAMP-response element binding protein (CREB), interferon gama (IFNγ), transforming growth factor B1(TGFB1), tumor necrosis factor alpha (TNFα), cyclooxygenase 2 (COX2), inducible nitric oxide synthase) iNOS, endothelial nitric oxide synthase (eNOS).
